# Troxerutin affects nephropathy signaling events in the kidney of type-1 diabetic male rats

**DOI:** 10.22038/AJP.2021.18875

**Published:** 2022

**Authors:** Rana Keyhanmanesh, Gholamreza Hamidian, Hajie Lotfi, Zohre Zavari, Monireh Seyfollahzadeh, Afsane Ghadiri, Mehdi Ahmadi, Farzad Bahari, Fariba Mirzaei Bavil

**Affiliations:** 1 *Tuberculosis and Lung Diseases Research Center, Tabriz University of Medical Sciences, Tabriz, Iran *; 2 *Emergency Medicine Research Group, Tabriz University of medical sciences, Tabriz, Iran*; 3 *Department of Physiology, Faculty of Medicine, Tabriz University of Medical Sciences, Tabriz, Iran*; 4 *Department of Basic Sciences, Faculty of Veterinary Medicine, University of Tabriz, Tabriz, Iran*; 5 *Drug Applied Research Center, Tabriz University of Medical Sciences, Tabriz, Iran*

**Keywords:** Diabetes, Troxerutin, miRNA192, Nephropathy, TGF-β, SIP1

## Abstract

**Objective:**

Nephropathy is known to be the leading cause of kidney failure in diabetic patients. Troxerutin, as a flavonoid component, could provide a novel protective strategy in the prevention of diabetic nephropathy. A large number of reports on the salutary effects of troxerutin inspired us to investigate its effect on the nephropathy signaling events (i.e., expression of TGF-β, miRNA192, and SIP1) in type-1 induced diabetic rats.

**Materials and Methods:**

50 male Wistar rats were divided into 5 groups including control group, sham group treated with troxerutin for 4 weeks, diabetic group induced by streptozotocin (STZ) injection, DI group including insulin-treated diabetic animals and DT group treated with troxerutin. Ultimately, rat kidneys were extracted, and the level of miR-192 (using qPCR), transforming growth factor-beta (TGF-β), and smad interacting protein 1 (SIP1) using an ELISA kit, was measured.

**Results:**

The level of TGF-β and miRNA192 significantly increased in the diabetic group. However, their expression levels decreased following the administration of troxerutin and insulin (p<0.05) compared to control group. SIP1 was down-regulated in the diabetic group, whereas a spike in the expression levels was observed after troxerutin administration compared to control and troxerutin groups (p<0.05). However, no significant difference was found in the effects of insulin and troxerutin on the level of miR-192, SIP1, and TGF- β.

**Conclusion:**

According to the previous literatures, during the progression of nephropathy, TGF-β represses SIP1 (the repression region in the collagen gene) by increasing the expression of miR-192. Ultimately, in this study, diabetes led to up-regulation of TGF-β while troxerutin proved to have a protective effect on the kidney by increasing SIP and lowering miR-192 levels.

## Introduction

Diabetes is a serious chronic disease worldwide and is anticipated to affect 700 million people by 2045 with an ever-rising prevalence. Common acute and chronic complications of diabetes include hypoglycemia, ketoacidosis, coma, cardiovascular disorders, neuropathy, retinopathy, and nephropathy (Østergaard et al., 2019 ▶; Vidyasagar, 2019 ▶). Among these complications, diabetic nephropathy is a serious complication in diabetic patients (Type 1 and 2), that could lead to end-stage renal disease and dialysis in 25-40% of the diabetic cases. Several risk factors are involved in the development of diabetic nephropathy including high blood glucose levels, long-term diabetes, hypertension, obesity, and dyslipidemia. Antidiabetic and antihypertensive medications, lipid-lowering drugs, and healthy lifestyles could help to manage many of these risk factors and reduce the chance of the disease progression (Tziomalos and Athyros, 2015 ▶). 

In the pathogenesis of diabetic nephropathy, the role of activated transforming growth factor-β (TGF-β) has been recognized. TGF-β acts through autocrine and paracrine pathways instigating the signaling chain by stimulation of corresponding receptors. Ultimately, the process leads to dysfunction of the mesangial cell by regulating synthesis of extracellular matrix (Braga et al., 2014 ▶). TGF-β accumulates in mesenchymal cells during the development of nephropathy and affects the production of extracellular matrix proteins such as collagen I and II. In addition, TGF-β could decrease the expression of E-box repressor (δEF1) in collagen genes (Kato et al., 2004 ▶). 

Several studies have focused on the utilization of microRNA biomarkers for fast and early diagnosis of nephropathies (Chien et al., 2016 ▶). microRNAs are small single-stranded RNAs containing 19-15 nucleotides that typically bind to the 3' untranslated region (3'-UTR) of target mRNAs, and regulate various biological events in disease progression (Wojczakowski et al., 2019 ▶; Müller et al., 2020 ▶). Moreover, increased levels of miR-192, which is one of the main renal miRNAs, have been determined in diabetic nephropathy. On the other hand, it has been reported that this miRNA could negatively impact smad-interacting protein 1 (SIP1), which is another E-box repressor (Tayel et al., 2020 ▶; Al‑Kafaji and Al‑Muhtaresh, 2018 ▶). Interestingly, high levels of TGF-β followed by up-regulation of miR-192 leads to decreased SIP1 expression (Al‑Kafaji and Al‑Muhtaresh, 2018 ▶). It has been confirmed that high levels of blood glucose in diabetic patients induce cytokine production, particularly TGF-β (Fan et al., 2019 ▶). 

Recently, various properties of troxerutin, also known as vitamin P_4_ which is a bioflavonoids derivative, have been reported, including its anti-oxidant, anti-cancer, and anti-diabetic effects (Raja et al., 2019 ▶; Thomas et al., 2019 ▶). Regarding the molecular pathways involved in nephropathy, in this study, we aimed to determine probable protective effect of troxerutin against nephropathy and investigate its mechanism of action on the TGF-β/miR-192/SIP1 pathway. According to a previous study (Fan et al., 2009 ▶) and our results, the potential protective effect of troxerutin on the maintenance of proper kidney function may provide therapeutic insights for diabetic patients.

## Materials and Methods

**Test subjects in the study**

This investigation was conducted as an interventional study. A total of 50 adult male Wistar rats weighing 200-250 g were purchased from the animal’s house of Tabriz University of Medical Sciences and were acclimatized for a week. Five groups of rats (10 rats per group) were included in the study, whose interventions are summarized in Table 1. 

**Table 1:** The rats in each group of the study and related interventions

**Animal groups**	**Intervention**	**Ref **
Control	Nothing	-
Sham (T)	Received troxerutin (150 mg per kg), once daily, for 4 weeks by oral gavage	-
Diabetic (D)	Induction of diabetes by intraperitoneal injection of 50 mg/kg streptozotocin (STZ)	
Diabetic with insulin (DI)	Received NPH insulin (4-6 units), once daily, for 4 weeks	
Diabetic with troxerutin (DT)	Received troxerutin (150 mg/ kg), once daily, by oral gavage for 4 weeks	

A single dose (50 mg/kg) of streptozotocin STZ (Sigma), dissolved in water, was injected to rats. After three days of injection the onset of diabetes was confirmed by a blood glucose level exceeding 250 mg/dl. In the next phase, troxerutin (dissolved in water) and insulin were administered. Bodyweight and blood glucose of rats were monitored weekly. A glucometer was used to measure the glucose levels of the rats` tail blood. Four weeks after the intervention, rats were anesthetized using ketamine (50 mg/kg, Sigma) and xylazine (5 mg/kg, Sigma). After extraction, the right kidneys were stored at -70˚C supplied by liquid nitrogen, until the next experimental procedure.

All procedures were carried out in accordance with the Guide for the Care and Use of Laboratory Animals of the National Institutes of Health (NIH; Publication No. 85-23, revised 1985). This study was approved by Tabriz University of Medical Sciences Ethical Committee (Ethics approval No. IR.TBZMED.REC. 1396.854).

**Evaluation of miR-192 level using qPCR**

Approximately 100 mg of kidney tissue was ground and homogenized in 700 µl of TRIzol reagent (Roche). The prepared supernatant was placed on ice for 10-15 min. Then, chloroform (200 µl) (Merck, Germany) was added and samples were put on ice for 10 min. After gentle inversion, samples were centrifuged at 12000 rpm, for 15 min at 4˚C. The upper bluish aqueous phase was transferred into RNAase-free tubes. Cold isopropanol (1 ml) was added to each tube. The tubes were then incubated for 20 min. After centrifugation, the supernatant was removed and the RNA plate was washed by ethanol 75% (1 ml) (Merck, Germany). The obtained RNA plates were air-dried and filled with diethyl pyrocarbonate (DEPC) water (40 µl) (Cinnagen, Iran). Finally, a short heating step at 60˚C was performed for 15 min in order to RNA re-solubilization. The concentration and quality of RNA were analyzed by measuring its absorbance (OD) at 260/280 nm using a Nanodrop spectrophotometer and 1% agarose gel electrophoresis. 

Complementary DNA (cDNA) synthesis was performed using 1 μg of RNA using Thermo scientific Revert Aide kit and thermal cycler T100tm (Bio RAD Co. USA). Expression of miRNA level including miRNA 192, and miRNA191 (as reference miRNA) was measured with SYBR Green Master Mix (Roche, Mannheim, Germany) with specific primers (see Table 2). The fold change was calculated according to the Pfaffl method (Pfaffi MW, 2001 ▶)

**Evaluation of TGF-β and SIP1 level using ELISA kits**

Smad-interacting protein 1 (SIP1) ELISA Kit (MyBioSource, Inc. Catalog Number: MBS908010) and TGF-β1 ELISA kit (MyBioSource, Inc. Catalog Number: MBS824788) were used to measure the level of SIP1 and TGF-β1, respectively. The OD of ELISA test results were read by a plate reader at 450 nm.

**Statistical analyses**

All data are reported as mean±SEM. The ANOVA test followed by the *post hoc* Tukey test was performed using GraphPad Prism 4 software (GraphPad Software, San Diego, USA). If p values were less than 0.05, the differences between groups were considered statistically significant. 

**Table 2:** miRNA primers sets were used in the qPCR

Target Sequence	**Accession Number**	**Gene Name**
CTGACCTATGAATTGACAGCC	MIMAT0000449٭	*rno-miR-* *192*
CAACGGAAUCCCAAAAGCAGCUG	MIMAT0000440٭	*rno-miR-191*
٭ www.mirbase.org		

## Results

**The effect of troxerutin on miRNA192 expression level in the kidney of diabetic rats**

**Figure 1:**
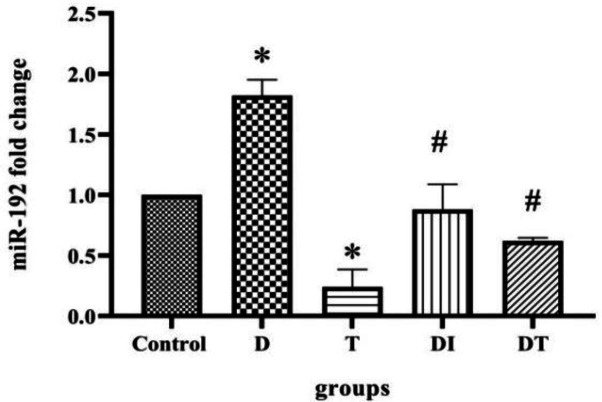
The effects of troxerutin on expression level of miR-192 in the kidneys of type 1 diabetic rats. Each bar represents the mean±SEM (n=10), ⁎p<0.001 in comparison with the control group; #p<0.0001 in comparison with the diabetic group. (D: diabetic, T: Received troxerutin, DT: Diabetic treated with troxerutin, and DI: diabetics that received insulin).

As depicted in Figure 1, in the diabetic group, the level of miR-192 was significantly increased compared with control group (p=0.0004). However, troxerutin produced a negative effect on miR-192 expression level compared to the diabetic group (p<0.0001). In the DI and DT groups, a diminished level of miR-192 (p<0.0001) was found, compared to the diabetic group. This was attributed to the effect of insulin and troxerutin. Overall, there was no difference between the groups that received insulin or troxerutin in terms of miR-192 expression. 

**The effect of troxerutin on TGF**-**β level in the kidney of diabetic rats**

**Figure 2:**
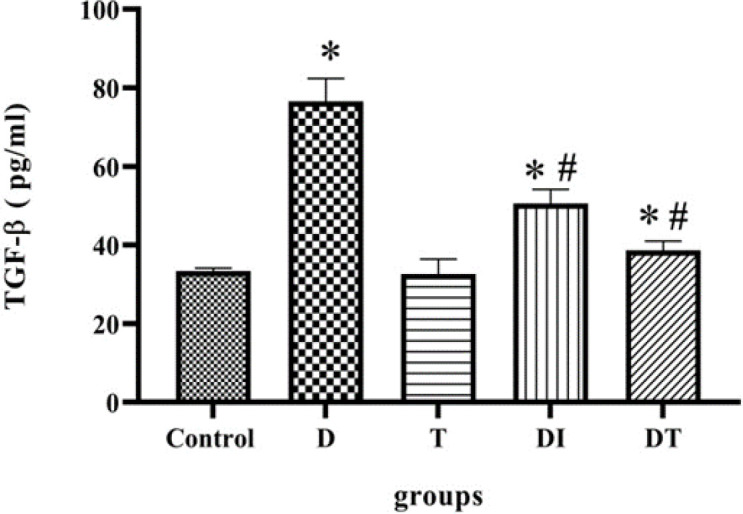
The effects of troxerutin on TGF-β level in the kidneys of type 1 diabetic rats. Each bar represents the mean±SEM (n=10), ⁎p<0.0001 in comparison with the control group; # in comparison with the diabetic group (D vs I: p<0.0001, D vs DI: p=0.0002, and D vs DT: p<0.0001) (D: diabetic, T: received troxerutin, DT: Diabetic treated with troxerutin, and DI: diabetics that received insulin).

The analysis showed that in diabetic groups, the level of TGF-β was higher, and statistically significant when compared with other groups (p<0.0001). Both insulin or troxerutin could reduce the expression level of TGF-β compared to the diabetic group. Nevertheless, between DI and DT groups, no difference was found in TGF-β level (p=0.16). In addition, the effect of troxerutin was negligible compared to the control group (Figure 2).

**The effect of troxerutin on SIP1 level in the kidney of diabetic rats**

**Figure 3:**
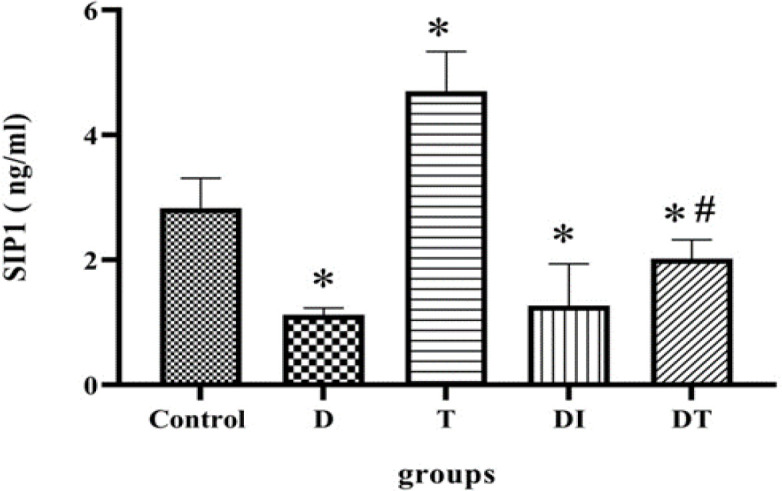
The effects of troxerutin on SIP1 level in the kidneys of type 1 diabetic rats. Each bar represents the mean±SEM (n=10), ⁎p<0.05 in comparison with the control group; # in comparison with the troxerutin group (D vs T: p<0.0001, T vs DT: p=0.0028). (D: diabetic, T: Received troxerutin, DT: Diabetic treated with troxerutin, and DI: diabetics that received insulin)

A significantly low level of SIP1 was reported in the diabetic group (D vs T: p<0.0001). However, administration of troxerutin led to a rise in SIP1 level, in comparison with the control group (p=0.03). Moreover, insulin and troxerutin increased SIP1 level in DI and DT groups, respectively. It was speculated that troxerutin effect was slightly stronger than insulin (T vs DI: p<0.0001, T vs DT: p=0.0028). However, it should be noted that there was no significant difference between insulin and troxerutin effect (Figure 3).

## Discussion

According to our findings, the levels of TGF-β and miR-192 were high in diabetic rats. Administration of insulin and troxerutin led to a decline in their levels, albeit with no statistical differences. Troxerutin had a negative effect on the miR-192 level in the sham group, however, no such effect on the TGF-β level was observed. Moreover, the SIP1 level was lower in the diabetic group; Administration of troxerutin in the sham group resulted in relatively higher levels of this protein. In the diabetic animals, expression of SIP1 was decreased by administration of insulin and troxerutin (with non-statistical differences).

In diabetic patients, high blood glucose and ketotic by-products could stimulate TGF-β expression leading to kidney damage. In nephropathy, collagen levels increase in the kidney tissue. Key factors involved in collagen formation could be considered for preventing nephropathy and hindering the progression of the disease. TGF-β stands out as a prominent factor, because it stimulates renal cell hypertrophy and formation of extracellular matrix (ECM) proteins (such as collagen I and fibronectin) as the hallmarks of renal disorders (Chang et al., 2016 ▶). It has been found that an elevation in TGF-β level results in the thickening of capillary membranes and proliferation of mesangial cells. Gewin et al. in 2020 discussed the biosynthesis pathway of several proteins including angiotensin II, reactive oxygen species (ROS), thrombospondin-1 (TSP-1), matrix metalloproteinases (MMPs) (MMP-2 and MMP-9), and hexosamine are involved in diabetes-induced TGF-β activity (Gewin et al., 2020 ▶). Considering the role of TGF-β in diabetic nephropathy, modification of this pathway by bioflavonoids such as troxerutin could have therapeutic value in an area of research that is remained vibrant. In our study, the diabetic groups exhibited a high level of TGF-β. Troxerutin was able to reduce high levels of TGF-β significantly, compared to the control group. Kato et al. (2007) ▶ explained the role of miR-192 in diabetic nephropathy in the presence of high levels of TGF-β (Kato et al., 2007 ▶). In addition, miRNAs have been previously recognized as biomarkers of different diseases. The potential of miR-192 and miR-173 as diabetic nephropathy biomarkers, has been evaluated by Al‑Kafaji and Al‑Muhtaresh (2018) ▶. In line with these reports, the levels of miR-192 and TGF-β were higher in the diabetic group, and according to our findings, the role of the investigated miRNA with nephropathy symptoms was highlighted.

In mesangial cells, the expression of collagen gene is regulated by TGF-β through different molecular mechanisms including mitogen-activated protein kinases (p38 and ERKs) and E-box elements (Kato et al., 2007 ▶). It has been demonstrated that TGF-β could down-regulate the δEF1 and SIP1 as an E-box repressor which regulates the collagen gene. In the context of nephropathy, the function/target of abundant miRNAs in the kidney (such as miR-192, miR-194, miR-204, and miR-215) has gained a lot of attention. Since SIP1 was identified as a target of miR-192, the low level of SIP1 in the diabetic group might confirm the crosstalk between high levels of TGF-β and miR-192, with low levels of SIP1 in kidney tissue. 

Regarding the relevance of these factors to the development of nephropathy, application of troxerutin could be considered a promissory novel approach in the management of this condition. Moreover, acceptable safety, negligible adverse effects, and multipotent biological activities of troxerutin are confirmed. In addition, a previous study reported the protective effect of troxerutin on kidney disorders (Fan et al., 2005 ▶). 

Liu et al. (2010) reported the antioxidant potential of troxerutin, and its effect on lowering ROS levels and NADPH oxidase activity. They concluded that troxerutin had protective effects on kidney tissue recovering from d-gal-induced injury. The thesis implied that troxerutin could improve renal function, by reducing histopathologic alterations and decreasing ROS production and DNA oxidative damage (Liu et al., 2010 ▶). In another study, the positive effects of troxerutin in subjects treated with cisplatin were confirmed which was attributed to the subtle regulation of malondialdehyde, blood urea nitrogen and serum creatinine levels, glutathione peroxidase, and superoxide dismutase activities. Furthermore, troxerutin also alleviated necrosis, degeneration, vacuolization, and cast of tubular cells (Dehnamaki et al., 2019 ▶). 

In our study, troxerutin was able to reduce TFG-β and miR-192 level, and increase SIP1 level in the diabetic group. However, in the sham group, troxerutin presented no effect on TGF-β level. Considering the contradictory results, other associated molecular mechanisms should be investigated.

As a conclusion, it has been determined that different miRNAs participate in the progression of diabetic nephropathy. In diabetic conditions, upregulation or downregulation of these miRNAs suppresses renoprotective genes. Our results indicated the protective role of troxerutin on kidney in a rat model of diabetes, mediated by increased SIP1 and lowered miR-192 level as an important miRNA in the development and progression of nephropathy. Future studies for evaluation of troxerutin effects on collagen level and ECM proteins are necessary to introduce troxerutin as a protective natural component suitable for the prevention of nephropathy.

## Conflicts of interest

The authors have declared that there is no conflict of interest.
